# Deep Proteomic Profiling of Human Stem Cell-Derived Microglia: Insights Into Inflammatory and Alcohol-Induced Reactivity

**DOI:** 10.21203/rs.3.rs-7983368/v1

**Published:** 2025-11-17

**Authors:** Tiara Wolf, Jessica Wohlfahrt, Jennifer Guergues, Stanley M. Stevens

**Affiliations:** University of South Florida; University of South Florida; University of South Florida; University of South Florida

**Keywords:** microglia, alcohol, hiPSC, neuroimmune, proteomics

## Abstract

Alcohol use disorder (AUD) is associated with significant neuroimmune dysregulation, with microglia emerging as a potential pathological driver of neurotoxicity. However, the molecular landscape driving this alcohol-induced reactivity has yet to be uncovered, especially in the context of human microglia. Here, we comprehensively characterized global-scale molecular changes of human stem cell-derived microglia (iMGLs) after exposure to ethanol and inflammatory mediators lipopolysaccharide (LPS) and TNFα using mass spectrometry-based proteomics. KOLF2.1J hiPSCs were differentiated to iMGLs utilizing commercially available kits and treated with multiple ethanol exposure paradigms, LPS, and TNFα. Proteomic profiles were assessed by data-independent acquisition (DIA) mass spectrometry, followed by a Luminex cytokine assay and a functional phagocytosis assay. Ethanol produced distinct, temporally dependent proteomic changes that differed markedly from the robust proinflammatory responses induced by LPS and TNFα. Cytokine measurement confirmed an overall lack of proinflammatory responses of iMGLs treated with ethanol in contrast to the elevated levels in LPS- and TNFα-treated cells. However, Ingenuity Pathway Analysis and flow cytometry revealed an increase in phagocytosis of iMGLs treated with ethanol for 48 hours. Our findings demonstrate that ethanol exposure induces distinct and temporally dependent molecular phenotypes in human iMGLs, which differ markedly from the robust proinflammatory responses elicited by LPS and TNFα. The novel pathways identified in this study will provide new insights into the human-specific response to ethanol and emphasizes the importance of utilizing human-specific models for neuroimmune interactions in AUD.

## INTRODUCTION

Many pathophysiological outcomes can arise from alcohol use disorder (AUD), including detrimental effects to multiple organ systems such as the brain. In 2023, an estimated 28.9 million people aged 12 and older in the U.S. reported having an AUD within the past year (1). These effects on the brain are driven by numerous, complex molecular mechanisms in a cell-type-specific manner; however, the neuroimmune response, which is partly modulated by microglia, has emerged as a pathological driver in the brain of AUD patients (2, 3). The microglial response to alcohol is a complex relationship that holds significance for our understanding of alcohol-related neurotoxicity and neurodegeneration as well as related molecular adaptations that result in behavioral and other phenotypic abnormalities. Microglia are the resident macrophages of the central nervous system (CNS) and contribute to neural homeostasis through functions such as phagocytosis of synapses and apoptotic cells and the release of cytokines and growth factors. Alcohol exposure induces a range of microglial phenotypes that are highly context dependent and not adequately described by M1 and M2 activation states ((4) and reviewed in (5)). Recent findings from our lab and others indicate mouse microglia exposed to alcohol display a phenotype that is not entirely proinflammatory (6–8), despite reports of brain-wide neuroinflammation associated with alcohol exposure ((3) and reviewed in (5, 9)). These findings emphasize the phenotypic diversity and complexity of the role microglia play in the neuroimmune response to alcohol and highlights the need for deep phenotypic characterization to understand the specific molecular adaptations and their implications for neurotoxicity and neurodegeneration in AUD patients.

*In vivo* approaches of alcohol exposure provide valuable insights into these molecular and cellular perturbations that occur in the brain upon alcohol exposure; however, there are inherent differences between rodent and human microglia, limiting the translational potential of findings obtained from rodent studies. A recent transcriptomic study found mouse microglia exhibit a lower expression level of several genes associated with Alzheimer’s disease when compared to humans, highlighting the limitation of phenotyping endogenous mouse microglia (10). Additionally, challenges in data interpretation are driven by species and strain-specific differences. The frequent use of C57BL mice exemplifies these limitations, as this strain carries a mutation in the *nnt* gene with implications in macrophage biology (11), calling into question the potential genotypic influence on microglial phenotype. Even further, two distinct lineages within the C57BL strain (6J and 6N) exhibit unique transcriptomic profiles in response to ethanol exposure (12), complicating our understanding of the effects of alcohol exposure. These species-specific differences in microglial biology and response to alcohol underscore the need for research approaches that incorporate human-specific microglia to enhance our understanding in the context of more translational findings.

To address these limitations, we utilized a well-characterized reference human induced pluripotent stem cell (hiPSC) line to generate human microglia, providing a standardized platform that overcomes both the species-specific constraints of rodent models and the variability inherent to clinically-derived hiPSCs. Differentiation of human induced pluripotent stem cells (hiPSCs) to microglial-like cells (iMGLs) (13) offers the potential to provide new insights into the mechanisms underlying alcohol-induced neuroinflammation. Therefore, we aimed to establish a reproducible and translationally relevant model using iMGLs and subsequently characterize the model’s global-scale molecular changes and reactivity to inflammatory agents and ethanol *in vitro*. Using commercially available kits, hiPSCs were differentiated to iMGLs over the course of 36 days, followed by treatment with ethanol and inflammatory mediators LPS and TNFα. Our phenotyping results indicate iMGLs exhibit unique phenotypic profiles when exposed to different models of alcohol exposure, and especially when compared to other inflammatory mediators, highlighting the unique temporal and model-specific mechanisms of alcohol-induced microglial reactivity.

## METHODS

### Cell culture

KOLF2.1J cells were purchased from The Jackson Laboratory (Bar Harbor, ME) and thawed into mTeSR Plus medium (STEMCELL Technologies, Vancouver, CA) supplemented with RevitaCell at 10 μM (Gibco, Waltham, MA). The cells were maintained in mTeSR Plus medium on Geltrex-coated (Gibco) 6-well plates at 37 °C with 5% (v/v) CO_2_. Aggregate passaging of the cells using ReLeSR (STEMCELL Technologies) was performed weekly, and cells were replated at a split ratio of 1:10 prior to differentiation to expand the stock. All cells utilized were < passage 10. iPSCs underwent assessment of morphology and sterility prior to differentiation experiments. Characterization of genotype through whole genome sequencing, genetic stability, and confirmation of pluripotency were completed prior to cell line distribution by JAX (Pantazis et al., 2022). Identification of markers associated with the undifferentiated state were additionally confirmed by mass spectrometry proteomics. Karyotype analysis was performed via the KaryoStat+ service (LifeTechnologies, Carlsbad, CA) prior to experimentation.

### Cell differentiation

hiPSCs were differentiated to hematopoietic progenitor cells (iHPCs) and microglia-like cells (iMGLs) using the STEMdiff Hematopoietic and STEMdiff Microglia Differentiation kits (STEMCELL Technologies), respectively. Briefly, iPSCs were passaged with ReLeSR and resuspended in mTeSR Plus. Sixty cell aggregates 100–200 μm in diameter were plated in each well of a Geltrex-coated 12-well plate, resulting in approximately 20 adhered colonies per well after incubation overnight. Media was replaced with differentiation media provided in the Hematopoietic kit as directed for 12 days. On the final day, iHPCs were collected and plated at a density of 2 × 10^5^ cells per well of a 6-well plate coated in Geltrex in 2 mL Microglial Differentiation medium, while extra iHPCs were cryopreserved in CryoStor (STEMCELL Technologies) prior to storage in liquid nitrogen. One half of the starting medium volume (e.g., 1 mL) was added to the cells every 2 days for 24 days to differentiate to iMGLs. Cells were transferred to a fresh Geltrex-coated 6-well plate on Day 12 in 2 mL Differentiation media to prevent overflow and refresh media.

### Immunocytochemistry

On the final day of the Microglia Differentiation kit, the iMGLs were transferred to a 6-well plate containing 3 glass coverslips coated in Poly-D-Lysine (Gibco) in media from the STEMdiff Microglia Maturation kit (STEMCELL Technologies) and allowed to incubate at 37 °C with 5% (v/v) CO_2_ overnight. At time of collection, the cells were fixed in 4% PFA for 15 minutes at RT, blocked in 0.5% donkey serum and 0.1% Tween20 in PBS (PBS-T) for 1 hour at RT, and stained with rabbit anti-IBA1 (Invitrogen, Waltham, MA, PIMA536257) at a 1:500 concentration overnight at 4°C. The coverslips were then washed thoroughly with PBS-T three times at half-hour intervals and stained with goat anti-rabbit AlexaFluor 488 secondary antibody (Invitrogen, A32731) at a 1:1000 concentration for 1 hour at RT. The coverslips were washed again with PBS-T prior to mounting using a DAPI mounting solution (Invitrogen, P36935). The slides cured overnight at 4°C followed by analysis and imaging on a Nikon T2 confocal microscope.

### Luminex Cytokine Assay

Cytokines from cell supernatant collected from the 24-hour treatment groups (control, 50 mM ethanol, TNFα, and LPS) were analyzed in both biological and technical triplicate using a Human Luminex Discovery Assay 4-plex for IL-27, IL-1β, IL-1α, and SPP1 (R&D Systems). Briefly, frozen cell culture supernatant was thawed and diluted 1:2 with kit-provided buffer. 50 μL of each sample, prepared standard, or blank was plated in the provided 96-well plate. Microparticles were then added to each well and incubated on an orbital plate shaker at 800 rpm for 3 hr at RT. Using a magnetic adapter, the plate beads were washed three times with kit-provided buffer before adding Biotin-Antibody and incubating again for 1 hr. The wash step was repeated, followed by incubation with Streptavidin-PE for 30 min at RT. The wash step was repeated for a final time, the beads resuspended in buffer, and the plate analyzed on a Luminex MAGPIX instrument. A standard curve was generated using a 5-parameter logistic curve-fitting method on xPONENT (version 4.2) to calculate cytokine concentrations, with a minimum microparticle count of 50 per region. Statistical comparisons were performed by one-way ANOVA followed by a Tukey post-hoc test.

### Microglial treatment

At the conclusion of the Microglia Differentiation kit, the iMGLs in the 24 hr group were transferred to a new Geltrex-coated 6-well plate (3 × 10^5^ cells/well) in 2mL Microglia Maturation media and allowed to incubate overnight. Cells were treated with Microglia Maturation media (control) or media supplemented to achieve a final concentration of 50 mM ethanol, with and without evaporative compensation as described previously (Rath et al., 2022), 50 ng/mL mouse TNFα, or 5ng/mL LPS and incubated for 24 hr. Cells in the 48 hr group were first transferred to a 12-well plate (1 × 10^5^ cells/well) in 1 mL Microglia Maturation media and incubated overnight. A similar approach was utilized for the 48-hour treatment with 50 mM ethanol with compensation; however, a media change was incorporated at the 24-hour mark to facilitate the withdrawal effect in the ethanol without compensation group. 20 μL of cell culture supernatant was collected from each sample at indicated time points for ethanol concentration measurement using the Pointe Scientific kit. At collection time, cell culture supernatant was collected and stored at −80 °C. The cells were detached by trituration and washed twice by centrifugation with ice cold DPBS (−/−) at 300 × g for 5 minutes at 4 °C. Cell pellets were snap frozen in liquid nitrogen and stored at −80°C until further processing for mass spectrometry analysis. Three biological replicates were collected for each treatment.

### Flow cytometry

At the conclusion of the STEMdiff Hematopoietic kit, Microglia Differentiation kit, and phagocytosis assay, cells were collected by trituration, counted using a hemocytometer, and washed twice via centrifugation with DPBS (−/−) at 300 × g for 5 minutes at 4 °C. For marker characterization, iHPCs were stained with fluorophore-conjugated CD34, CD43, and CD45 and iMGLs were stained with fluorophore-conjugated CD45, CD11b, and TREM2. iMGLs in the phagocytosis assay were stained with only fluorophore-conjugated CD11b. The cells were incubated with the antibodies protected from light for 30 minutes on ice followed by a DPBS (−/−) centrifugation wash. DAPI live-dead stain was added, and the cells analyzed on a BD FACS Melody. Singlets were identified by plotting FSC-H against FSC-A. Cells were gated on SSC-A against FSC-A. A minimum of 20,000 events was collected per sample.

### Phagocytosis Assay

The iMGLs (n=4, 1 ×10^5^ cells each) were subjected to the 48 hr models of ethanol exposure and control treatment in a 12-well plate. 1 hr before the 48 hr mark, frozen fluorescent HiLyte^™^ Fluor 488 amyloid-β (1–42) (Aβ) (AnaSpec) was resuspended in DMEM and incubated at 37 °C to promote aggregation. Thirty min before the 48 hr mark, half of the replicates were treated with 10 μM cytochalasin D (CyD), an inhibitor of actin filamentation and phagocytosis. At 48 hr, Aβ was added to each well to result in a final concentration of 0.5 μM Aβ in the cell culture media. One well was not treated with either CyD or Aβ to serve as a background for flow cytometry gating. The cells were then incubated at 37 °C for 1 hr before collection and analysis by flow cytometry. Four wells were collected for each condition, for a total of 24 samples analyzed plus 1 background sample. The cells were then collected, washed, fixed in paraformaldehyde for 15 minutes, and analyzed by flow cytometry as described previously. Singlets and cells were gated as described previously. FITC-Aβ positivity was determined by calculating the upper 99.5^th^ percentile of FITC intensity of all CyD+Aβ-treated groups in R (flowCore v 2.18). The calculated FITC threshold of 7251.358 was applied to all samples, limiting the false-positive rate in negative controls to 0.5%.

### Mass spectrometry-based proteomics and data analysis

Frozen cell pellets were processed for LC-MS/MS proteomics using an iST kit (PreOmics GmbH, Planegg/Martinsried, Germany) according to manufacturer instructions, as previously described by our group (8). In the 24 hr treatment groups (control and EtOH, and control, LPS, and TNFα) n=3 samples of approximately 3 ×10^5^ cells were processed per condition, while the 48 hr treatment groups (control, EtOH compensation, and withdrawal) were processed n=4 of approximately 1 ×10^5^ cells. Total protein was quantified using a microplate-based assay (Pierce 660 Protein Assay) and samples normalized prior to digestion. The 24 hr treatment group samples were analyzed by diaPASEF on a timsTOF Pro instrument with a nanoElute UHPLC (Bruker, Billerica, MA); similarly, the 48 hr treatment group samples were analyzed by diaPASEF on the same instrumentation but with an upgraded nanoElute 2 UHPLC (Bruker). Samples were heated and ionized using a CaptiveSpray ion source with column oven heated to 50°C containing an Aurora Ultimate CSI UHPLC reversed-phase C18 column (25 cm × 75 μm i.d., 1.7 μm C18, IonOpticks, Fitzroy, Australia). Mobile phases A (0.1% formic acid in water) and B (0.1% formic acid in acetonitrile) were used in a 45-min gradient of 2–25% B (total run time of 60 min including a 37–80% B ramp up to clean and prepare the column for the next sample). DIA-PASEF scan mode was utilized within an ion mobility range of 0.7– 1.40 1/K_0_ [V·s/cm^2^] spanning 250–1425 m/z, resulting in a 1.48 s estimated cycle time. Both ion mobility and m/z calibrations were performed linearly using three ions at 622 m/z, 922 m/z, and 1222 m/z (Agilent, Santa Clara, CA).

DIA data were analyzed in DIA-NN (v. 1.9.1) (14) using a predicted library generated from the UniProt Homo sapiens database (82861 entries). A label-free quantification (LFQ) approach was utilized with match-between-runs (MBR) applied, using a 1% protein and precursor FDR cutoff with additional run-specific 1% FDR filter at the protein level, along with selection of reannotate, single pass mode, genes, quantUMS (high precision), RT-dependent, and IDs RT &IM profiling.

Statistical testing was conducted in Perseus (v.2.1.3.0) (15) as previously described (8). In brief, the pg_matrix.tsv output file was formatted by removing contaminants and ensuring identification columns allowed subsequent Ingenuity Pathway Analysis (IPA) gene and/or protein ID mapping. Microglial proteome datasets were filtered where the protein groups that did not have at least 3 valid values in at least one variable group were removed. Missing values in the remaining protein groups were replaced via the imputation function (values selected from the normal distribution option) with a width of 0.3 and downshift of 1.9 to fit the lower abundance of the Gaussian curve in each replicate of each condition. A Welch’s t-test was performed on the 24-hour treated control and ethanol proteome comparisons, while a 1-way ANOVA with p-value cutoff of < 0.05 followed by a post hoc Tukey test with FDR of 0.05 were performed for the 24-hour inflammatory mediator and 48-hour ethanol proteome comparison separately to control for multiple comparisons within each data set. Finally, GO and Kegg term annotations were added through Perseus to each final output before exporting to excel for further sorting and filtering.

In Excel, average ratios were calculated for each relevant variable comparison for all datasets. In the 24-hour treated control and ethanol proteomic comparison, in addition to the Welch’s t-test cutoff of p < 0.05, an additional |z-score| cutoff of > 1 was employed with the purpose of controlling the false discovery rate (FDR) and increasing confidence for further validation (16). Additionally, proteins that had one comparison group with 2 or more missing original (pre-imputed) values have purple font while those with all original values missing in one of the comparison groups have their cells highlighted red. Protein groups that met statistical cutoffs in all data sets were then uploaded to Ingenuity Pathway Analysis (IPA) for further bioinformatic evaluation. Subsequent predicted downstream functional pathways generated were filtered for activity z-score cutoff > 2 (predicted activation) or < −2 (predicted inhibition) as well as enrichment p-value < 0.05 (Fisher’s Exact Test with Benjamini-Hochberg correction).

## RESULTS

### iPSCs are differentiated to iMGLs using a kit-based approach

Prior to experimentation, iPSCs underwent quality control assays, including sterility, morphology, karyotyping, and identification of markers indicating the undifferentiated state as recommended by the ISSCR (17) (Fig. S1). The cells were found to be negative for mycoplasma (Fig. S1A) and displayed morphology characteristic of iPSCs (Fig. S1B). Mass spectrometry-based proteomics identified 54 proteins associated with the undifferentiated state, including POU5F1 and SOX2 (Table S1). Karyotype analysis, shown in Fig. S1C, identified a gain mutation on chromosome 3 affecting the following genes: FHIT, PTPRG, FEZF2, CADPS. Similar karyotypic abnormalities have been previously found in the KOLF2.1J line (18), but reportedly do not compromise the model in neurological disease applications (19). Following these quality control analyses, the iPSCs were used to establish a Master Bank and Working Bank of cells, from which the following experiments were derived.

**Figure 1:**
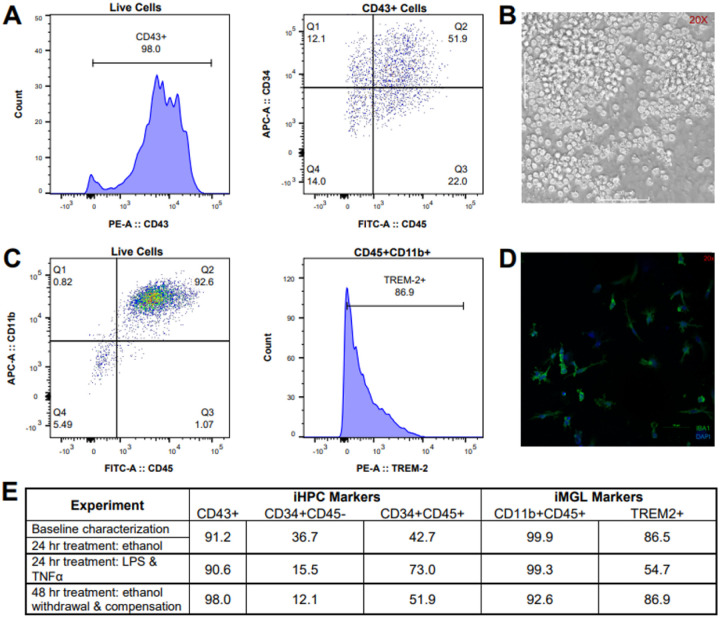
Characterization of hematopoietic and microglial differentiation. (A) The first representative flow cytometry histogram shows the percentage of live iHPCs expressing CD43. The second representative dot plot displays the percentage of CD43+ cells also expressing CD34 and CD45. (B) Representative image of differentiated iHPCs in culture taken at 20X. Scale bar represents 100 μm. (C) Representative dot plot displaying the percentage of live iMGLs expressing CD11b and CD45. The second representative histogram plot displays the percentage of CD11b+CD45+ cells also expressing TREM2. (D) Representative confocal microscopy image taken at 60X of iMGLs stained with anti-IBA1 (green) and DAPI nuclear stain (blue). Scale bar represents 10 μm. (E) Flow cytometry data from all experiments conducted is shown. All rounds of differentiation displayed high levels of key markers (>90% CD43 for iHPCs and >90% CD11b+CD45+ and >50% TREM2+ for iMGLs).

iPSCs were differentiated to iMGLs using two commercially available kits from STEMCELL Technologies, which are based on previous publication (13). The iPSCs are first differentiated to a mesodermal state then hematopoietic progenitor cells (iHPCs) over the course of 12 days in the STEMdiff Hematopoietic kit. An average of 5 × 10^5^ cells were collected from each well of the 12-well plate and an aliquot analyzed by flow cytometry for CD43, CD45, and CD34 surface markers. As several rounds of differentiation were necessary to complete all experimental steps, this process was repeated for all iHPC batches and >90% CD43+ expression was confirmed for each population, indicating successful differentiation per kit benchmarks. Additionally, the CD43+ population was further characterized as CD34+CD45- or CD34+CD45+ which corresponds to multipotent and myeloid-skewed lineage (20), respectively, which both have the capacity to differentiate to microglia. Representative flow cytometry plot of iHPCs are shown in Fig. 1A, with the marker percentages of each analyzed population shown in Fig. 1E. Additionally, iHPCs showed expected morphology in cell culture, with cells phase-bright, floating, and round (Fig. 1B). Following confirmation of successful differentiation by marker expression, hematopoietic cells were resuspended in a cryoprotectant solution (CryoStor, STEMCELL Technologies) at a density of 1 million cells/mL and stored in liquid nitrogen until further experimentation.

Frozen hematopoietic cells were rapidly thawed and plated at a density of 22,000 cells/mL for culturing in the STEMdiff Microglial Differentiation kit for 24 days. One day before the conclusion of the kit, a portion of cells was replated in a 12-well plate with Poly-D-Lysine-coated glass coverslips for 24 hours for immunocytochemistry analysis while the remaining cells were left undisturbed. The next day, cells were collected for proteomics and flow cytometry and the coverslips were fixed and prepared for confocal imaging. Flow cytometry of iMGLs for expression of CD11b, CD45, and TREM2 was used to assess differentiation. All iMGL batches were analyzed and found to have >90% CD11b+CD45+ cells, with >50% of these CD11b+CD45+ cells also expressing TREM2, indicating successful differentiation per kit benchmarks. Representative flow cytometry plots of iMGLs are shown in Fig. 1C. Immunocytochemistry staining of the iMGLs for IBA1 and DAPI, shown in Fig. 1D, revealed ramified morphology characteristic of homeostatic microglia (21, 22). Of note, the kit reports up to 20% of the iMGLs may display an ameboid morphology, associated with reactive microglia. Few iMGLs were found to be ameboid in appearance when cultured on Poly-D-Lysine plates and imaged using the confocal microscope; however, iMGLs cultured on Geltrex for the mass spectrometry experiments had an overall more rounded appearance. A comparison of morphology differences due to culturing conditions is shown in Fig. S3.

**Figure 2:**
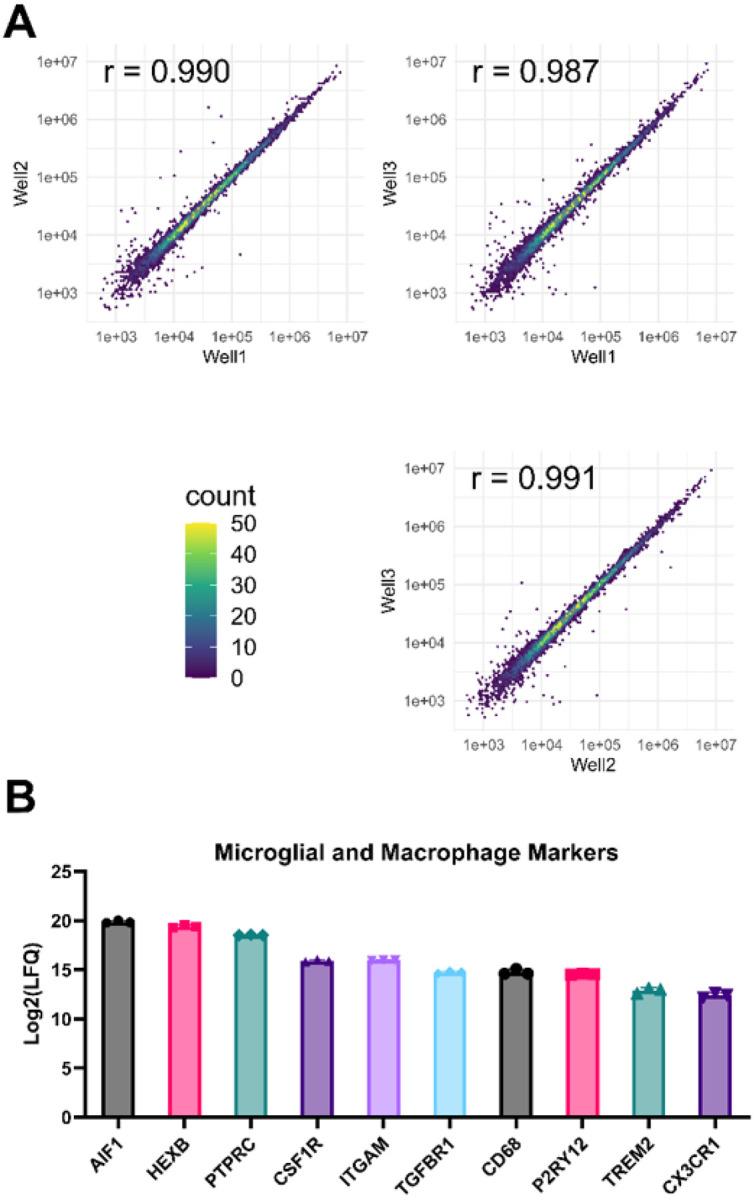
Mass spectrometry characterization of baseline iMGLs. Three wells of a 6-well plate with 3 ×10^5^ iMGLs per well were collected and processed for mass spectrometry-based proteomics. (A) Representative correlation plots and Pearson r values between each well are shown, showing high reproducibility of differentiation. (B) Log2-transformed Label-Free Quantification (LFQ) values of several microglial and macrophage markers.

Untreated iMGLs were then analyzed by mass spectrometry-based proteomics for identification of baseline protein expression. Three wells of a 6-well plate (3 × 10^5^ cells/well) were collected, processed by iST (PreOmics), and analyzed on a timsTOF Pro instrument. 6,994 proteins were identified from the samples at an FDR of 1%. Correlation plots of protein identification between each well display Pearson r values ranging from 0.987 to 0.991, indicating high reproducibility of differentiation (Fig. 2A). Additionally, proteins associated with microglia and macrophages, including those involved in migration, phagocytosis, and recognition, were identified in the baseline proteome (Fig. 2B).

### Mass spectrometry of treated iMGLs reveal ethanol and inflammatory exposure produces unique proteomic responses

**Figure 3:**
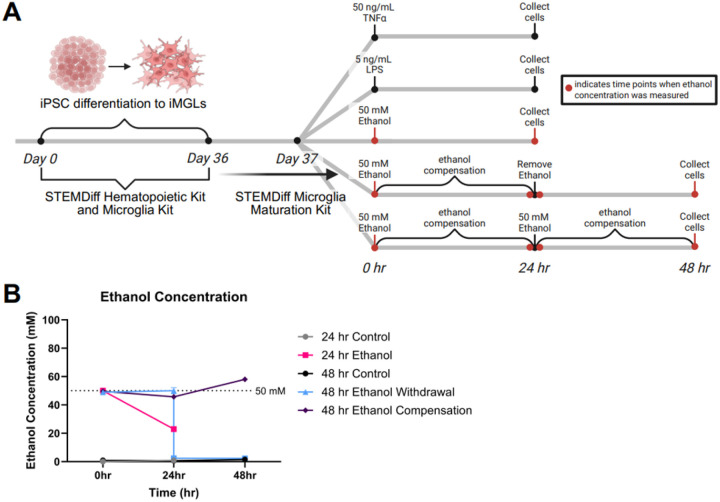
Ethanol and inflammatory mediator exposure strategy and ethanol concentration *in vitro*. (A) iPSCs were differentiated to iMGLs over the course of 36 days in the STEMDiff Hematopoietic kit (12 days) and the STEMDiff Microglia kit (24 days). On the final day of the Microglia kit, iMGLs were transferred to the STEMDiff Microglia Maturation kit and remained in this media until collection. 24 hr after being transferred to the new media, treatments were applied to the cells. The timing and exposure strategy are illustrated, with red dots on each timeline indicating when 20 μL media was collected for ethanol concentration measurement. (B) The concentration of ethanol from sampled media was determined using a Pointe Scientific BAC measurement kit. All control samples had concentrations within normal physiological levels (<10 mM ethanol). The ethanol compensation group had an average concentration of 51.15 mM ± 3.645 SEM across all measurements. Figure made with Biorender.com.

Following initial characterization, differentiated iMGLs were then cultured in the STEMdiff Maturation kit, which allows for maintenance of iMGLs for up to 10 days. After 24 hours in the Maturation media, iMGLs were treated with several models of ethanol exposure and inflammatory mediators LPS and TNFα. The cells were treated with kit media as control or kit media supplemented to achieve a final concentration of either 50 mM ethanol, 50 ng/mL mouse TNFα, or 5 ng/mL LPS once added to each well. LPS was employed as a positive comparison of the proinflammatory response given the relevance of TLR4-based signaling in alcohol-induced neuroinflammatory processes (23–25). TNFα similarly produces a proinflammatory response, and is also implicated in the neuroinflammatory cascade seen in patients with AUD ((3) and reviewed in (26)). The employed ethanol models include a 24-hour exposure with evaporation (EtOH 24 hr Acute), a 48-hour exposure with evaporative compensation to maintain a constant ~50 mM ethanol concentration (EtOH 48 hr Compensation), and a 48-hour withdrawal model, consisting of 24 hours of 50 mM ethanol with compensation, and 24 hours with no ethanol (EtOH 48 hr Withdrawal). Exposure strategies are illustrated in Fig. 3A. Cell culture media was sampled at the beginning of treatment (0 hr), at collection time (24 or 48 hr), and in the case of the 48 hr models, before and after the media change at 24 hr. The ethanol concentration of these samples was measured by the Pierce BEC kit, with results shown in Fig. 3B. The 24 and 48 hr control samples had an average concentration below the normal reference range of 2.1 mM. The 24 hr ethanol acute group had a concentration of 22.9 ± 0.8 mM at collection time, indicating approximately half the starting ethanol was cleared or evaporated from the cell culture media. The 48 hr ethanol compensation group maintained an average concentration of 51 ± 6.3 mM. Lastly, the 48 hr ethanol withdrawal group had a concentration of 50 ± 7.3 mM at the 24 hr mark, which decreased to 2.3 ± 1.6 mM after changing to fresh media, and ended at 2.3 ± 0.7 mM at collection time. These samples were slightly above the normal reference range of 2.1 mM, likely representing slight residual ethanol from the media change.

**Figure 4:**
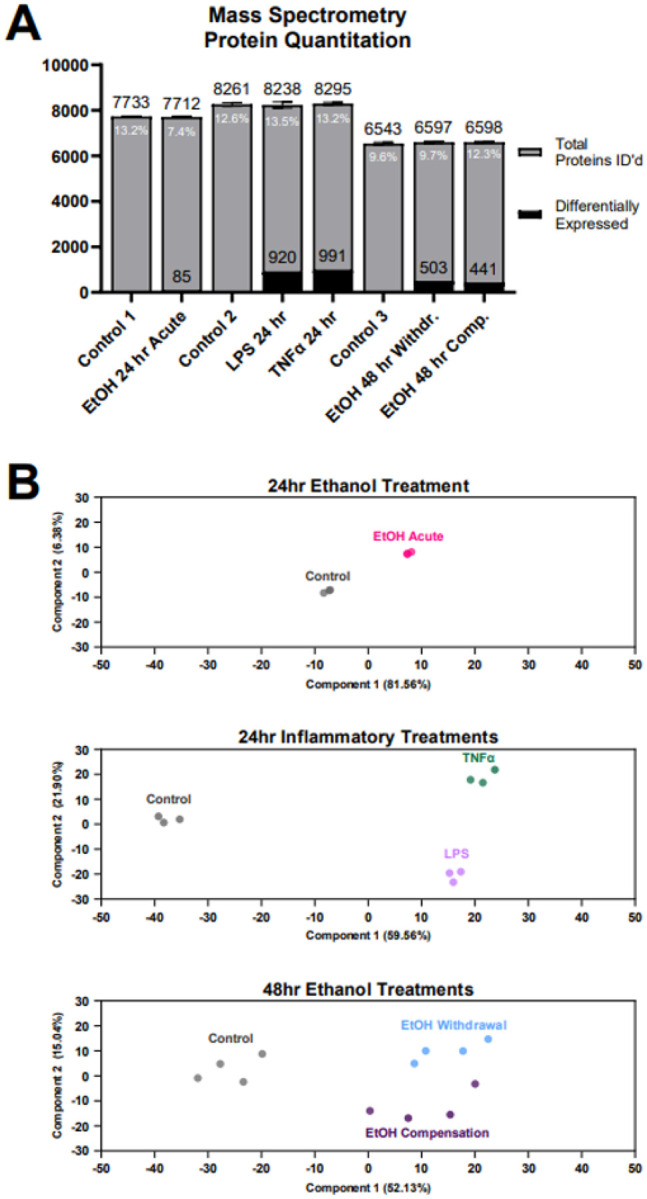
Mass spectrometry analysis of treated iMGLs. (A) The total number of identified proteins (gray) and number of differentially expressed proteins (black) in each treatment group when compared to its respective control are displayed in the bar chart. Median CV% is reported within each corresponding bar. Data bars represent mean ±SEM. (B) Principal Component Analysis (PCA) plots illustrate sample clustering of log2-transformed LFQ protein intensities.

The collected iMGLs were then processed for mass spectrometry as previously described. Fig. 4A displays the total number of proteins identified at 1% FDR of each group. Differentially expressed proteins were identified through the comparison of each treatment group with its corresponding control group. In the 24 hr ethanol acute group, 85 differentially expressed proteins were identified, whereas the 24 hr LPS and TNFα groups had 920 and 991 differentially expressed proteins, respectively. The 48 hr ethanol compensation and withdrawal groups had 441 and 503 differentially expressed proteins, respectively. Both the global proteome and differentially expressed proteins are shown in Table S2–7. Analysis of the differentially expressed proteins revealed unique proteomic profiles for each treatment group. Principal Component Analysis (PCA) was conducted for each experimental group to assess variance and sample clustering of the log2-transformed Label-Free Quantification (LFQ) protein intensities in each dataset (Fig. 4B). Each replicate of a given treatment clustered closely within the plot. The treatment groups clustered widely across PC1 when compared to the controls and further separated along PC2 depending on treatment type.

### Cytokines and signaling molecules are differentially expressed and released with ethanol and inflammatory mediator exposure

**Figure 5:**
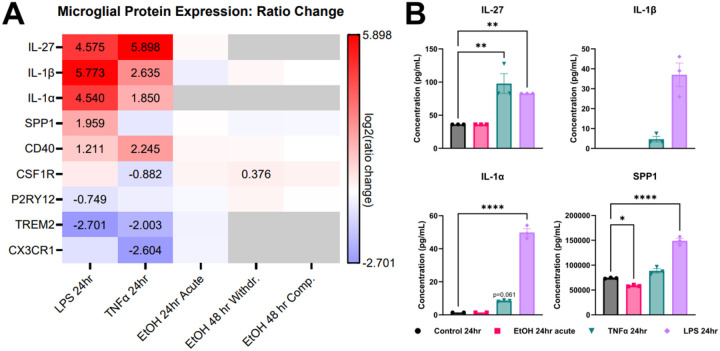
Treated iMGL marker and signal expression. (A) Log2(ratio change) values of key microglial proteins measured by mass spectrometry are shown for each treatment versus its control. Boxes with color but no value indicate the protein was measured but did not meet the significance cutoff, while gray boxes indicate the protein was not measured in the dataset. The first four proteins, IL-27, IL-1β, IL-1α, and SPP1, represent cytokine and signaling molecules, while the other proteins represent microglial and macrophage markers. (B) Secreted cytokines from the 24 hr exposure experiments were measured from the cell culture media using a Luminex Multiplex cytokine assay. LPS and TNFα induced a significant increase in cytokine expression versus control in most measured cytokines, while ethanol produced no significant difference versus control except in SPP1, where a significant decrease was observed. Significance was determined by one-way ANOVA with Tukey post-hoc. Data bars represent mean ±SEM.

Several cytokine and signaling molecules, as well as key microglial and macrophage markers, were found to be significantly differentially expressed in the proteomics datasets of the LPS and TNFα treatment groups when compared to control. The cytokine and signaling molecules were comprised of interleukin (IL)-27, IL-1β, IL-1α, and SPP1, also known as osteopontin. IL-27 is a pleiotropic cytokine with functions in both pro- and anti-inflammatory processes and has been shown to be expressed in response to TNFα (27) and LPS (28). IL-1a and IL-1b are both associated with the myeloid differentiation primary response 88 (Myd88)-dependent inflammatory response following activation of the inflammasome (29). SPP1 is a mediator of several inflammatory processes in macrophages, including migration, cytokine release, and phagocytosis (30). Differential expression of these proteins from TNFα and LPS treatment, identified by mass spectrometry and displayed in Fig. 5A, suggest a proinflammatory phenotype of the microglia. However, in almost all ethanol-treated groups, these same proteins failed to meet the significance threshold for differential expression or were not detected at all.

These proteomic findings were validated using a Luminex multiplex cytokine assay. Supernatant collected from the samples treated with 24 hr ethanol acute, LPS, and TNFα (n=3) were assayed in triplicate for concentrations of IL-27, IL-1β, IL-1α, and SPP1. LPS treatment led to a significant increase in IL-27 (2.29-fold), IL-1α (33.62-fold), and SPP1 (2.01-fold), compared to control as determined by one-way ANOVA with Tukey post-hoc. TNFα led to a significant 2.7-fold increase in IL-27 and trending (p=0.061) 5.7-fold increase of IL-1α. IL-1β was not detected in either control or ethanol groups but was detected in the TNFα (4.675 pg/mL) and LPS (37.04 pg/mL) groups. In all comparisons of control versus ethanol, no significant differences were detected, except for SPP1 where a 1.26-fold decrease was observed. These measured secreted proteins mirror the patterns observed in the proteomics data where, generally, TNFα and LPS treatment led to a significant increase in these proteins while treatment with ethanol failed to recapitulate this upregulation.

### Ethanol treatment for 48 hours enhances phagocytosis of amyloid beta

**Figure 6:**
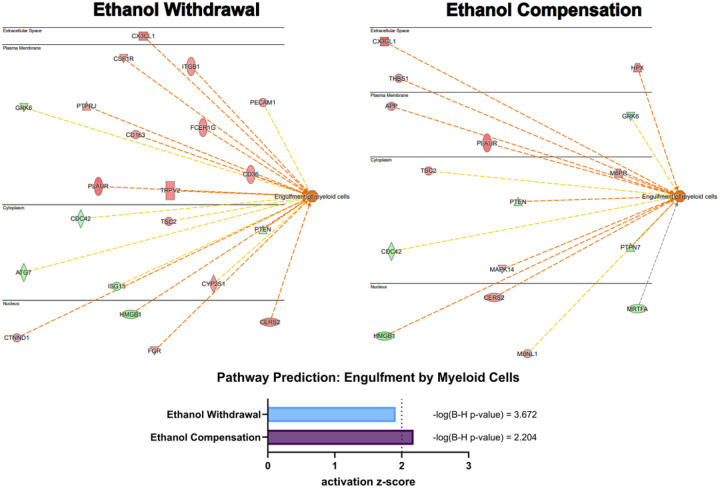
Bioinformatic prediction of iMGL phagocytosis in the 48 hr treatment groups. Ingenuity Pathway Analysis (IPA) was employed for prediction of pathway inhibition and activation of iMGLs that underwent the 748 hr ethanol withdrawal and compensation models. Each node represents a protein involved in the pathway, with the color overlay representing an experimentally measured increase (red) or decrease (green) in protein expression. The bar chart displays the calculated z-score of predicted activation of each treatment along with Benjamini-Hochberg corrected p-values. Activation of engulfment by myeloid cells had a trending prediction with ethanol withdrawal (z-score=1.910) and significant prediction in with ethanol compensation (z-score=2.178). Significant pathway activation is defined as z-score > 2.

Bioinformatic analysis with Ingenuity Pathway Analysis (IPA) of the 48 hr treated iMGLs revealed predicted activation of hydrogen peroxide and reactive oxygen species (ROS) metabolism and phagocytosis, two major functional pathways of microglia, with both ethanol exposure groups. IPA calculated a z-score of predicted pathway activation for the Engulfment of myeloid cells pathway of 2.178 and 1.910 for ethanol compensation and withdrawal, respectively, with significant pathway activation defined as a z-score ≥ 2 (Fig. 6). A p-value following Benjamini-Hochberg correction of 2.204 and 3.672, respectively, were reported. While the z-score prediction was only trending for the ethanol withdrawal group, the upregulation of scavenging receptors CD36 and CD163 in the proteomics data is consistent with enhanced phagocytic activity (31, 32). Of note, CSF1R was also upregulated with the ethanol withdrawal group, which has been previously reported by our group with ethanol treatment, and is involved in phagocytosis (33). Predicted activation of the Metabolism of hydrogen peroxide (z = 2.518 and z = 3.959, respectively) and Biosynthesis of ROS (z = 2.169 and z = 3.679, respectively) observed in both ethanol-treated groups are consistent with altered oxidative metabolism induced by ethanol (34).

**Figure 7:**
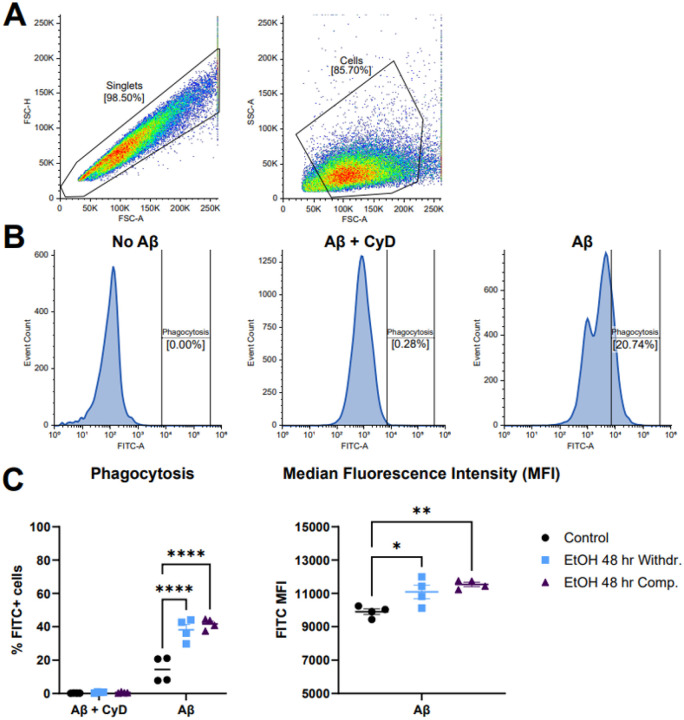
Flow cytometry phagocytosis assay of 48-hour ethanol-treated cells. (A) The gating strategy for excluding doublets, debris, and identifying cells is shown. (B) Representative phagocytosis plots of untreated control, Aβ and cytochalasin D (CyD) inhibitor, and Aβ-treated cells are shown. FITC-Aβ positivity was defined as the upper 99.5^th^ percentile of FITC intensity of all cells in the Aβ + CyD-treated groups. (C) Quantification of phagocytosis and Median Fluorescence Intensity (MFI) of the Aβ-treated groups are plotted. Both ethanol-treated groups had a significant increase when compared to control within the Aβ-treated group for phagocytosis (2-way ANOVA with Tukey post-hoc) and MFI (1-way ANOVA with Tukey post-hoc). Data bars represent mean ±SEM.

Given the relevance of microglial phagocytosis in the progression of neurodegeneration (35), this predicted activation was investigated using fluorescently-labelled amyloid beta (Aβ) followed by flow cytometry analysis. Analysis of fluorescent Aβ uptake by flow cytometry is shown in Fig.7. Fig.7A shows the gating strategy used to isolate single cells and exclude debris. FITC positivity was determined by gating at the 99.5^th^ percentile of all Aβ + CyD control samples, thus limiting false positives to 0.5%. All gates were applied consistently to all samples and representative plots are shown in Fig.7B. The average percentage of phagocytosing cells from the Aβ groups were 14.5% ± 7.5% for control, 38.2% ± 6.6% for ethanol withdrawal, and 41.7% ± 3.2% for ethanol compensation. Within the Aβ group, both the ethanol compensation and ethanol withdrawal groups had significantly higher phagocytosis than control (2-way ANOVA with Tukey post-hoc). Median Fluorescent Intensity (MFI) of positive cells in the Aβ-only groups were analyzed to determine the effect of ethanol on phagocytic capacity. A significant increase in MFI was observed in both ethanol withdrawal (11,092 ± 804) and compensation (11,545 ± 259), compared to control (9,906 ± 344) by 1-way ANOVA with Tukey post-hoc.

## DISCUSSION

Recent advances in techniques for global-scale characterization have highlighted limitations in the M1/M2 framework for describing microglial reactivity (36). Additionally, accumulating evidence has established rodent microglia differ substantially from human microglia at both baseline and in disease states (37–39). These species differences limit the translational applicability of findings from rodent-derived models, highlighting the growing need for human models to better capture microglial biology. In this study, we employed iPSC-derived microglia from the KOLF2.1J reference line to establish a reproducible and translationally relevant model to investigate global-scale molecular changes and reactivity to inflammatory agents and ethanol. Through deep proteomic profiling, flow cytometry, and functional assays, we demonstrate that iMGLs exhibit unique phenotypes in response to ethanol, especially when compared to inflammatory mediators LPS and TNFα. These findings both validate the utility of KOLF2.1J-derived iMGLs as a scalable and accessible platform for human microglial research and provide novel insight with direct relevance to human neuroimmune dysregulation in AUD.

We found the KOLF2.1J iPSCs were reproducibly differentiated to iMGLs across all experiments conducted for the study. All iMGLs utilized met the minimum expected marker expression of > 90% CD11b + CD45+, with > 50% of these CD11b + CD45 + cells co-expressing TREM2. CD11b and CD45 serve as microglial-specific markers within the CNS. TREM2 expression is particularly of interest, given its complex roles in abrogating and driving neurodegeneration (40), and its potential role in AUD (41). One limitation in the use of iPSCs is Copy Number Variants (CNVs), which may be introduced during culture or intrinsic to the cell line. We identified a gain mutation on chromosome 3 that has been previously associated with the KOLF2.1J line (18), impacting several genes. Of note, PTPRG is a reported tumor suppressor with roles in neuropsychiatric and inflammatory disorders (42). PTPRG may also play a role in microglia-neuron crosstalk in Alzheimer’s disease progression (43). This CNV did not grossly impact the cell line’s function in our chosen model or experimental design; however, this finding should be considered when comparing results between different iPSC lines. In terms of morphology, the differentiated iMGLs showed a ramified, or branched, appearance when cultured on Poly-D-Lysine coated plates and imaged using a confocal microscope. However, kit guidance reports up to 20% of iMGLs may display ameboid morphology. This rounded appearance was more prevalent in the iMGLs cultured on Geltrex, which may be due to the culturing conditions given iMGLs are only semi-adherent to the cultureware on Geltrex versus strongly adhered when cultured on Poly-D-Lysine. However, our proteomic and cytokine analysis of control cells showed profiles consistent with homeostatic microglia, such as the absence of IL-1α, indicating that this appearance was not related to reactivity.

Proteomic analysis of treated iMGLs found unique phenotypes associated with each treatment condition. Acute 24-hour exposure to LPS or TNFα induced a robust proinflammatory response, with over 900 differentially expressed proteins identified in each group. In contrast, the 24-hour ethanol treatment led to a subtle proteomic shift of only 85 differentially expressed proteins identified. These findings are consistent with our group’s previous proteomic studies reporting limited microglial responses to ethanol both *in vitro* and *in vivo* (6–8, 33, 44). Notably, several proinflammatory signaling proteins were identified in the LPS and TNFα groups but were either not detected or not significantly altered in the ethanol-treated groups. Extending the treatment window to 48 hours in the compensation and withdrawal models resulted in over 400 differentially expressed proteins; however, bioinformatic analysis with IPA did not predict an activation of canonical proinflammatory response pathways, instead suggesting an increase in hydrogen peroxide and ROS metabolism and phagocytosis. Taken together, these results demonstrate that human iMGLs respond to inflammatory stimuli and ethanol with distinct molecular phenotypes. The differential responses to LPS, TNFα, and ethanol are particularly relevant, given the involvement of TLR4 signaling and TNFα-mediated neuroinflammation in AUD (24, 45).

Microglial phagocytosis in response to ethanol has had mixed results in the literature, with some *in vitro* studies finding inhibition of phagocytosis after ≤ 24 hours (6, 46), while others found activation at ≥ 24 hours (23). *In vivo* studies of chronic alcohol consumption in rodents have found long-term use induces synapse loss (41, 47), implicating microglia as drivers of neuron loss and neurotoxicity. In a previous study of ethanol-treated rat microglia, CSF1R was found to be upregulated after 12 hours of 50 mM ethanol treatment (33), consistent with the homing phase of microglia described immediately prior to the phagocytosis phase (48). Here, we found our models of 48-hour ethanol exposure induced proteomic changes indicating an increase in phagocytosis as predicted by bioinformatic analysis with IPA, which then was confirmed functionally via increased uptake of fluorescent Aβ aggregates. These findings may suggest a temporal dependency of phagocytosis, whereby microglia may first decrease then increase their phagocytic capability with time. However, microglial function may be strongly influenced by genetic background, with a recent study utilizing stem cell-derived microglia showing differential effects of AUD Polygenic Risk Score on microglial phagocytosis and other cellular functions (49). To minimize donor variability and improve reproducibility, we employed the KOLF2.1J reference line that has been deeply characterized through whole-genome sequencing, several differentiation protocols, and has shown reliable CRISPR gene editing (50). Since the establishment of the KOLF2.1J line in 2022, other healthy reference iPSC lines have become commercially available, representing an area of further proteomic and functional characterization to determine the robustness and generalizability of ethanol-induced phenotypes across cell lines and polygenic risk.

In conclusion, our findings demonstrate that ethanol exposure induces distinct and temporally dependent molecular phenotypes in human iMGLs, which differ markedly from the robust proinflammatory responses elicited by LPS and TNFα. These results suggest that ethanol in this context does not activate proinflammatory pathways, but instead alters other functional processes, such as phagocytosis. By employing a model with high reproducibility of differentiation and translational relevance, this study provides new insights into the human-specific response to ethanol at a global proteomic scale. These findings lay the foundation for future work focusing on the dissection of microglia-neuron interactions in the context of AUD and emphasize the importance of utilizing human-specific systems to better model neuroimmune interactions in alcohol misuse.

## Supplementary Material

Supplementary Files

This is a list of supplementary files associated with this preprint. Click to download.

- S.Table1iPSCProteinGroupMatrix.xlsx
- S.Table27.xlsx
- 2024.04.1215.42.01FlUV.jpg
- GraphicalAbstract.docx
- SUPPLEMENTARYFIGURES.docx

## Data Availability

Mass spectrometry data have been deposited to the MassIVE repository with the dataset identifier MSV000099329. Reviewer account details: Username: MSV000099329_reviewer Password: iMGLproteomics
